# Non-invasive in vivo assessment of 11β-hydroxysteroid dehydrogenase type 1 activity by ^19^F-Magnetic Resonance Spectroscopy

**DOI:** 10.1038/s41598-022-18740-5

**Published:** 2022-09-29

**Authors:** Gregorio Naredo-Gonzalez, Rita Upreti, Maurits A. Jansen, Scott Semple, Oliver B. Sutcliffe, Ian Marshall, Brian R. Walker, Ruth Andrew

**Affiliations:** 1 University/British Heart Foundation Centre for Cardiovascular Science, University of Edinburgh, Edinburgh, EH16 4TJ Scotland, UK; 2 Edinburgh Imaging, Queen’s Medical Research Institute, 47 Little France Crescent, University of Edinburgh, Edinburgh, EH16 4TJ Scotland, UK; 3 Department of Natural Sciences, Faculty of Science and Engineering, Manchester Metropolitan University, Chester Street, Manchester, M1 5GD UK; 4 Centre for Clinical Brain Sciences, Chancellor’s Building, 49 Little France Crescent, University of Edinburgh, Edinburgh, EH16 4SB Scotland, UK; 5 Institute of Translational and Clinical Research, Newcastle University, Newcastle upon Tyne, NE1 3BZ UK

**Keywords:** NMR spectroscopy, Diabetes, Biomarkers, Endocrinology, Chemistry, Preclinical research

## Abstract

11β-Hydroxysteroid dehydrogenase type 1 (11β-HSD1) amplifies tissue glucocorticoid levels and is a pharmaceutical target in diabetes and cognitive decline. Clinical translation of inhibitors is hampered by lack of in vivo pharmacodynamic biomarkers. Our goal was to monitor substrates and products of 11β-HSD1 non-invasively in liver via ^19^Fluorine magnetic resonance spectroscopy (^19^F-MRS). Interconversion of mono/poly-fluorinated substrate/product pairs was studied in Wistar rats (male, n = 6) and healthy men (n = 3) using 7T and 3T MRI scanners, respectively. Here we show that the in vitro limit of detection, as absolute fluorine content, was 0.625 μmole in blood. Mono-fluorinated steroids, dexamethasone and 11-dehydrodexamethasone, were detected in phantoms but not in vivo in human liver following oral dosing. A non-steroidal polyfluorinated tracer, 2-(phenylsulfonyl)-1-(4-(trifluoromethyl)phenyl)ethanone and its metabolic product were detected in vivo in rat liver after oral administration of the keto-substrate, reading out reductase activity. Administration of a selective 11β-HSD1 inhibitor in vivo in rats altered total liver ^19^F-MRS signal. We conclude that there is insufficient sensitivity to measure mono-fluorinated tracers in vivo in man with current dosage regimens and clinical scanners. However, since reductase activity was observed in rats using poly-fluorinated tracers, this concept could be pursued for translation to man with further development.

## Introduction

**Figure 1:**
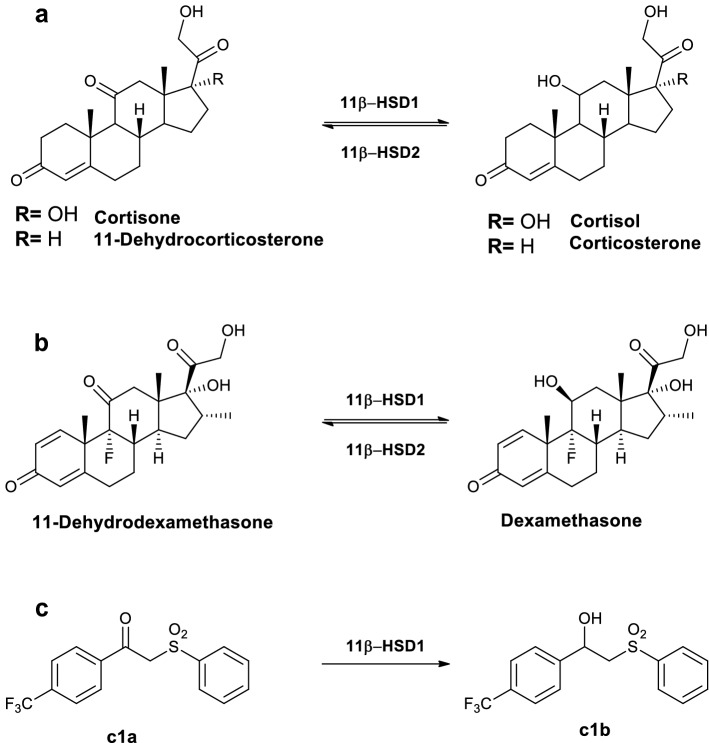
Metabolism of substrates by 11β-hydroxysteroid dehydrogenases. 11β-Hydroxysteroid dehydrogenase (11β-HSD) 1 reduces 11-keto steroids and non-steroidal tracers. **(a)** 11β-HSD1 converts inactive keto glucocorticoids cortisone or 11-dehydrocorticosterone into active hydroxy glucocorticoids, cortisol or corticosterone, respectively. 11β-HSD2 catalyses the reverse dehydrogenase reactions. **(b)** 11β-HSD1 and 11β-HSD2 also interconvert synthetic fluorinated steroids, 11-dehydrodexamethasone and dexamethasone. **(c)** The synthetic non-steroidal fluorinated compound **c1a** is also a substrate for 11β-HSD1, being converted to **c1b**.

Excessive activity of glucocorticoids results in many adverse effects including obesity, hyperglycaemia and cognitive impairment. The hypothalamic–pituitary–adrenal axis regulates glucocorticoid levels in the circulation but an additional level of control exists within tissues through local metabolism. 11β-Hydroxysteroid dehydrogenase type 1 (11β-HSD1) catalyses the conversion of inactive 11-ketosteroids into active 11-hydroxyglucocorticoids (Fig. 1a). This enzyme is expressed widely, including in liver, brain and adipose tissue^1^. In obesity, tissue-specific dysregulation of 11β-HSD1 results in increased enzyme activity in adipose tissue, with elevated local glucocorticoid concentrations and hence metabolic dysfunction^2^. With ageing, higher hippocampal 11β-HSD1 activity associates with more rapid cognitive decline in mice^3^. Genetic disruption or inhibition of 11β-HSD1 in mice lowers tissue glucocorticoid levels and protects against weight gain, hyperglycaemia^4,5^ and cognitive impairment^6,7^. In humans, a non-selective 11β-HSD inhibitor, carbenoxolone, improved metabolic and cognitive indices^8,9^, and selective inhibitors have progressed to Phase II clinical trials^10,11^. However, efficacy has been inconsistent and most drug development programmes have stalled. One reason for variable responses in patients is that adequate enzyme inhibition may not have been achieved in key target tissues. Many chemical series of selective 11β-HSD1 inhibitors exhibit species-specificity^12^ so the pharmacodynamics for clinical candidates cannot be reliably evaluated in pre-clinical models.

To date, whole-body 11β-HSD1 inhibition has been measured in humans by urinary cortisol metabolite ratios^11,13^ or during stable isotope labelled, tracer infusion^14^. Inhibition in liver has been assessed indirectly by generation of plasma cortisol or prednisolone respectively after oral cortisone or prednisone administration^13^. Enzyme activity in subcutaneous adipose tissue can be assessed ex vivo in biopsies^2^ and in vivo using microdialysis^9^ and glucocorticoid regeneration has been quantified in hepatic and adipose tissue specifically by tracer infusion combined with selective venous sampling^15^ and ex vivo by mass spectrometry imaging^16^. However, none of these tools assess nascent enzyme activity in vivo at a tissue level, and those allowing serial tissue-specific measurements (d_4_-cortisol infusion, microdialysis) are invasive and disturb the endogenous equilibrium. There is a need for pharmacodynamic tools to assess 11β-HSD1 non-invasively in vivo in humans, including in target tissues such as liver and brain.

Magnetic resonance spectroscopy (MRS) identifies molecules according to the resonance of specific nuclei, which is dependent upon their molecular and chemical environment. In combination with magnetic resonance imaging, MRS can quantify specific compounds in designated regions of interest within organs in vivo. The most common nuclei detected are protons (^1^H) and ^1^H-MRS has been used to detect abundant endogenous molecules^17,18^. The specificity of MRS is improved by measuring less common endogenous nuclei^19,20^ such as ^31^P or nuclei such as ^19^F, usually exogenous. ^19^F-MRS has been used successfully to study the distribution of fluorinated drugs^21–23^ in vivo in humans particularly in the oncology and psychiatry fields. Interest has also been paid to the potential of ^19^F-MRS to monitor metabolism of fluorinated drugs due to the change in chemical shift of ^19^F^24–28^ wrought by the biotransformation. ^19^F-MRS has been reported recently for detection of fluorinated steroids ex vivo in urinary extracts^29^, and in ocular tissues and fluids^30,31^ and here we assessed the potential of ^19^F-MRS for non-invasive in vivo measurement of 11β-HSD1 activity within liver, by differentiating fluorinated keto substrates and hydroxy products. Emphasis was placed on developing a protocol that minimised scanning time and tracer doses for pharmacodynamic use. The starting point was dexamethasone, a 9α-fluorinated steroid, licensed for human use, which is regenerated from 11-dehydrodexamethasone by 11β-HSD1 (Fig. 1b)^32,33^. To enhance sensitivity, potential poly-fluorinated substrates were screened, and one (Fig. 1c) progressed to proof-of-principle pre-clinical studies in vivo.

## Results

### Evaluation of monofluorinated tracers

#### In vitro evaluation of ^19^F-MRS of monofluorinated 11-dehydrodexamethasone and dexamethasone

The anticipated ability to discriminate 11-dehydrodexamethasone (substrate) from dexamethasone (product) by conventional chemical structure elucidation NMR (250 MHz ^1^H spectrometer) was confirmed in vitro showing different ^19^F resonance frequencies (Δδ_F_ 2.2 ppm) for the 9-fluorine atom according to whether the oxidation state of the carbon in the 11-position was determined by the presence of a ketone or hydroxy functionality (Supplementary Figure S1a).

**Figure 2:**
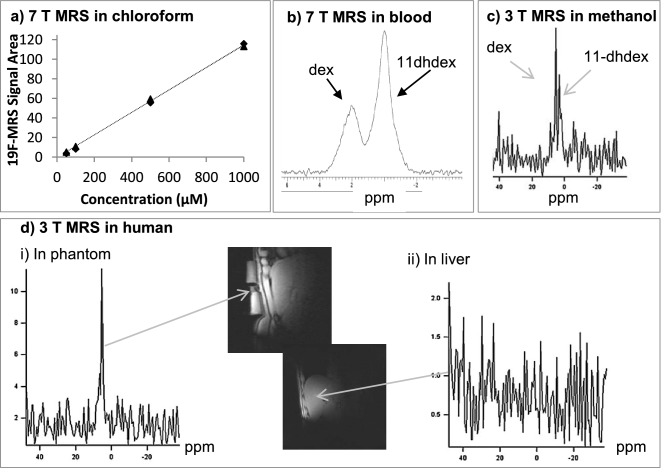
Evaluation of 11-dehydrodexamethasone as a tracer for 11β-HSD1 activity by ^19^F-MRS. **(a)** Linearity of ^19^F-MRS signal vs concentration (50–1000 µM) ketosteroid 11-dehydrodexamethasone (11-dhdex; triangles) and hydroxysteroid dexamethasone (dex; rhombi). **(b)** Broadening and overlap of peaks in whole blood biological matrix (5 mL) with 2.1 mg dex plus 4.2 mg 11-dhdex scanned on the Agilent 7T pre-clinical MR scanner with 300 repetitions and total scan time of 5 min. The spectrum is centred on the 11-dhdex peak, and the dex peak is 2.2 ppm apart. **(c)** Discrimination of dex and 11-dhdex is attenuated on the clinical Verio 3T scanner (10 mg each in 20 mL methanol). **(d)** (i) In healthy men, dex was detected in a phantom containing 10 mg dex placed within the coil next to the patient. (ii) Dex was not detected in the liver after oral administration of dexamethasone; a representative image is shown from a volunteer 10 min after a 12 mg dex dose.

Using a 7T small animal MR scanner, 11-dehydrodexamethasone and dexamethasone were also easily distinguished in solution in chloroform (Δδ_F_ > 2 ppm), closely matching the separation measured on the NMR spectrometer. Scanning conditions were optimised across a range of concentrations of 11-dehydrodexamethasone in chloroform. The shortest measured Repetition time (T_R_) that did not affect signal intensity was 0.5 s and linearity of the concentration/signal intensity relationship was maintained between 50 and 1000 μM (Fig. 2a) with scanning times of at least 100 s (with signal-to-noise (S/N) above 5 at the lowest point), (Supplementary Figure S2). The limit of detection as moles of fluorine (LOD_F_) was 0.250 μmol (98 μg of 11-dehydrodexamethasone) in chloroform. ^19^F-MRS signal intensities and indices of linearity of response of dexamethasone were the same as for 11-dehydrodexamethasone (Fig. 2a). Signal line width in chloroform was between 68 and 72 Hz across all concentrations. Increasing scanning times did not change signal areas but did improve sensitivity by substantial gains in S/N, so an interval of at least 400 s per scan was selected as a suitable duration for further in vivo pharmacodynamic measurements.

Whole blood was used as a biological matrix to mimic in vivo scanning with the 7T pre-clinical animal scanner. The optimal measured T_R_ was 1.0 s. 11-Dehydrodexamethasone and dexamethasone were scanned together at a 2:1 ratio. Both steroids were easily detected with peak maxima ≈ 2.2 ppm apart but signals were broader (Fig. 2b, dexamethasone 245 Hz, 11-dehydrodexamethasone 189 Hz) than in chloroform, which caused some overlapping of peaks. However, individual peak areas could still be calculated reliably using jMRUI processing (measured ratio 1.9:1). The LOD_F_ measured in blood in vitro was 0.625 µmol (250 μg of 11-dehydrodexamethasone)*.*

### In vivo ^19^F-MRS of 11-dehydrodexamethasone/dexamethasone in humans

Next the detection of dexamethasone and 11-dehydrodexamethasone signals was evaluated on a 3T clinical human scanner, requiring T_R_ of 1.5 s with 640 repetitions (16 min scan time) and providing a S/N of 2.5 and 5.2 with 2 and 10 mg of dexamethasone respectively. This indicated that LOD_F_ on the 3T scanner was suppressed by at least one order of magnitude compared to measurements performed at 7T. Discrimination of ^19^F-MRS signals of dexamethasone and 11-dehydrodexamethasone was also poorer on the 3T clinical scanner (Fig. 2c), compared to the data obtained with the 7T preclinical scanner.

Three healthy men were subsequently studied after oral administration of increasing doses of dexamethasone. The 10 min scans used fewer repetitions than the in vitro validation experiments (400 vs 640), aiming to achieve a more detailed pharmacokinetic profile of dexamethasone. A clear ^19^F-MRS signal was detected for dexamethasone in the phantom used to validate the scanning set-up (Fig. 2d(i)) but ^19^F-MRS signals for dexamethasone and 11-dehydrodexamethasone were not detected in vivo (Fig. 2d(ii)). The raw Free Induction Decay (FID) data for the 6 spectra were then combined (equivalent to 1 h long acquisition spectra) to compensate for the decreased number of acquisitions in each individual spectrum, but ^19^F-MRS signals for dexamethasone and 11-dehydrodexamethasone were still not detected. Quantitation of dexamethasone in plasma ex-vivo indicated a peak in circulating concentrations of around 170 μg/L. In plasma 11-dehydrodexamethasone was detected with peak concentrations of around 12 μg/L (≈0.43 and 0.03 µmol/L, respectively). It was concluded that fluorinated tracers held potential but that in vivo sensitivity in human using a 3T clinical scanner with standard product MRS acquisition sequences is insufficient to detect mono-fluorinated compounds, such as the pairing of dexamethasone and 11-dehydrodexamethasone, within an appropriate dose ranges. The use of polyfluorinated tracers was then investigated.

### Identification of polyfluorinated substrates of 11β-HSD1

**Figure 3:**
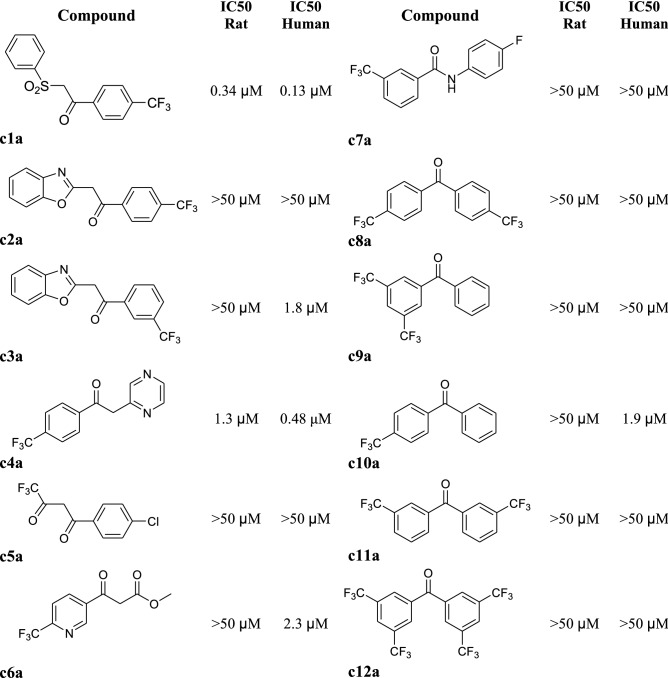
Screening putative polyfluorinated tracers for competition with cortisone for metabolism by 11β-HSD1. Inhibition of cortisone reductase activity by potential fluorinated keto tracers (**c1a**–**c12a**) thought likely to be substrates for 11β-hydroxysteroid dehydrogenase (11β-HSD 1) was evaluated in HEK293 stably transfected with human or rat *Hsd11b1.* IC_50_ values represent the mean of two experiments.

A library of potential preclinical tracers was studied, each of which had at least one carbonyl group that could potentially be reduced by 11β-HSD1 and contained 3 to 12 magnetically equivalent fluorine atoms (Fig. 3). The library was screened for inhibition of cortisone metabolism by human and rat 11β-HSD1. Compounds **c1a**, **c3a**, **c4a**, **c6a** and **c10a** had IC_50_s in the low μM range for human 11β-HSD1. Of these, only **c1a** and **c4a** showed a competitive interaction with rat 11β-HSD1 (Fig. 3), indicating differential inter-species tracer suitability. Compound **c1a** was selected for progression to proof-of-principle ^19^F-MRS studies in rat, since it had the lowest IC_50_ and was a known substrate for 11β-HSD1 ^34^. Synthesis of **c1a** stock and its reduced (hydroxy) from **c1b** was carried out in house (Supplementary Material).

### In vitro evaluation of ^19^F-MRS of polyfluorinated compounds c1a and c1b

**Figure 4:**
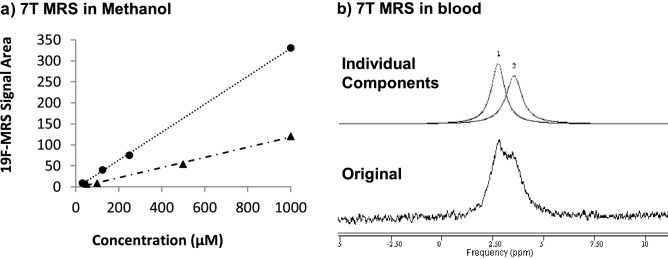
In vitro and ex vivo evaluation of compound **c1a** as a tracer for 11β-HSD1 activity by ^19^F-MRS.** (a)** Increased ^19^F-MRS signal from trifluorinated **c1a** (circles) vs monofluorinated 11-dehydrodexamethasone (triangles), measured in chloroform solution on the 7T pre-clinical scanner with T_R_ of 0.5 s, 800 repetitions and 400 s acquisition time. The ^19^F-MRS signal was linearly correlated with tracer amount (r^2^ > 0.99 for both **c1a** and 11-dehydrodexamethasone) and signal intensity increased linearly with numbers of fluorine atoms per molecule, as demonstrated by trifluorinated **c1a** giving a signal threefold higher than 11-dehydrodexamethasone. **(b)** Overlap of ^19^F-MRS peaks (upper panel) for **c1a** and **c1b** in blood and their resolution (upper panel) by jMRUI software using predefined peak ppm values for **c1a** (1) and **c1b** (2).

Using the 7T preclinical scanner, compound **c1a** in methanol was scanned across similar a concentration (31–1000 μM) range previously validated for the monofluorinated steroidal tracer and in similar rapid conditions (400 s per spectra, TR = 0.5 s) as monofluorinated dexamethasone/11-dehydrodexamethasone. ^19^F-MRS signal intensity was consistently three-fold higher for **c1a** than for dexamethasone or 11-dehydrodexamethasone at similar concentrations (Fig. 4a). LOD_F_ with S/N > 5 in these conditions for **c1a** was 0.470 μmoles (50 μg of **c1a**). Linearity and LOD of the **c1b** signal vs concentration/total fluorine content in the sample also matched that of **c1a** in the concentration range studied (Fig. 4a). Chloroform was also used as diluting solvent for *in-vitro* experiments with identical results to methanol (not shown).

Switching to blood as a matrix and maintaining the scan time at 400 s, the measured LOD_F_ was approximately four-fold higher than when in solvent. For example, for **c1b** LOD_F_ was 1.87 μmoles of equivalent fluorine (0.625 μmoles or 0.2 mg of **c1b**) with a S/N of 6.5. When **c1a** and its reduced metabolite **c1b** were scanned in rat blood ex vivo, the ^19^F-MRS signals observed were substantially broader than in organic solvents and the overlap between keto and hydroxy signals for **c1a**/**c1b** was extensive (Fig. 4b). The separation between signals closely matched the 0.6 ppm ^19^F-NMR signal difference (Supplementary Figure S1b) between **c1a** and **c1b** in CDCl_3_.

jMRUI AMARES processing was used for signal modelling and the accuracy of this procedure to measure changes in concentration at levels near the limit of detection (0.1–0.4 mg) was validated by comparing observed with expected peak area ratios from blood samples containing **c1a** and **c1b** (Table 1).

**Table 1:** Validation of spectral modelling of overlapping ^19^F-MRS peaks for **c1a** and **c1b **using jMRUI software.

c1a: c1b	^19^F-MRS signal area		^19^F-MRS signal ratio c1a:c1b	
mg: mg	c1a	c1b	Observed	Expected
0.4: 0	4033	–	n.a.	n.a.
0.4: 0.1	4261	1158	3.68	4
0.4: 0.2	3628	2177	1.66	2
0.4: 0.4	4125	4160	0.99	1

The proportions of peak areas of **c1a** (keto tracer) and **c1b** (hydroxy metabolite) in blood (4 mL) showed reasonable agreement with the expected ratio. 0.4 mg of **c1a** in blood was scanned alone and after addition of variable amounts of **c1b** at near their Limits of Detection (0.1–0.4 mg). Spectra were acquired with 800 repetitions (400 s). Signal areas are in arbitrary units. n.a. = not applicable.

### In vivo ^19^F-MRS of polyfluorinated compounds c1a and c1b in rat to detect 11β-HSD1 activity

**Figure 5:**
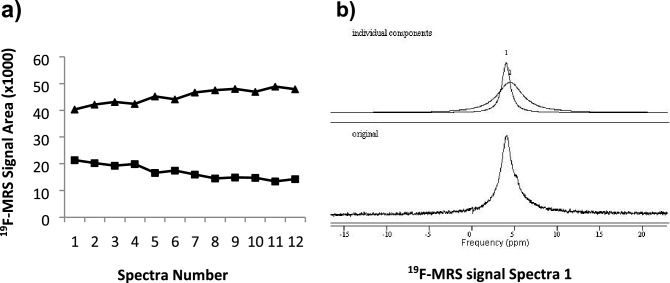
Ex-vivo (perfusion) and in-vivo conversion of keto compound **c1a** to hydroxy metabolite **c1b** detected by ^19^F-MRS scanning of liver in rat. (**a**) *Ex-vivo* scan of perfused liver. Signal areas versus spectrum on the serial scans of the pilot experiment, each for 400 s, beginning ~ 20 min after the end of a 30 min perfusion with **c1a** 100 µM solution. **c1b** was formed and accumulated rapidly during the perfusion. Generation of **c1b** from **c1a** continued to happen in the excised liver during scanning. (**b**) The ^19^F-MRS spectra reveals further broadening of peaks compared with scanning in blood (representative spectrum 1 and signal deconvolution shown), with the overlap of wide signals from tracer and metabolite seen across all timepoints.

Conversion of **c1a** into **cb1** could be detected following ex vivo hepatic perfusion of **c1a** (Fig. 5a). The total ^19^F-MRS signal measured from the excised liver remained constant over the spectral acquisition time. A change in the **c1a**:**c1b** ratio was measurable over time. jMRUI peak fitting suggested greater broadening of the ^19^F-MRS peaks in whole liver (Fig. 5b) than previously seen in blood.

**Figure 6:**
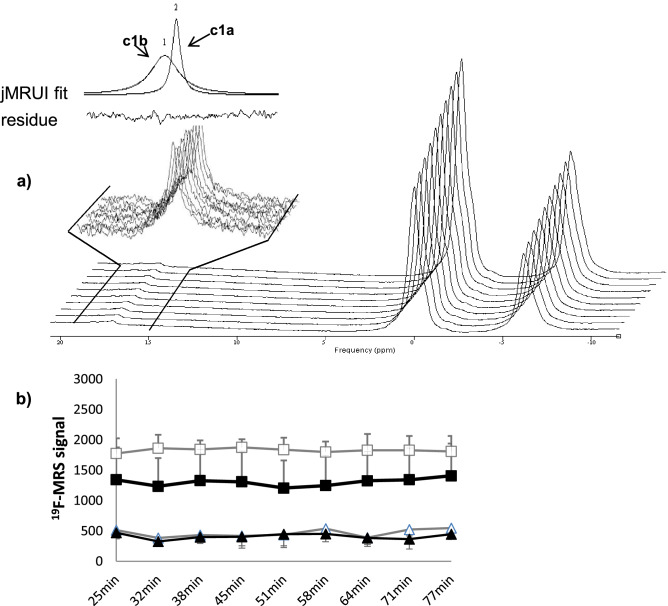
Effects of 11β-HSD1 inhibition on **c1a** and **c1b** in rat liver in vivo. **(a)** Sequential (front to back) in vivo ^19^F-MRS spectra in rat liver after gavage of 15 mg/kg of **c1a** post administration of a weight matched dose of vehicle. 800 repetitions (400 s) per spectrum were used to allow fine monitoring of signal change over time. ^19^F-MRS signal from **c1a** and **c1b** were detected (zoomed section on left hand side of spectra), with low in intensity compared to isofluorane peaks. **(b)** Peak areas for **c1a** and **c1b** measured by ^19^F-MRS after oral gavage of **c1a** in rats pre-treated with Merck 544 (MK544) selective 11β-HSD1 inhibitor (**c1a** black squares, **c1b** black triangles) or vehicle (**c1a** white squares, **c1b** white triangles). Data are mean ± SEM from n = 3 per group. The time shown is between **c1a** gavage and the start of the scan for each spectrum (aligned ± 2 min). Scan time per spectrum was 400 s (6 min 40 s). There were no statistically significant differences in amount of metabolite formed or ratio of substrate to product. However, by repeated measures ANOVA ^19^F-MRS signal of the substrate (**c1a**) on Merck 544 group was lower than for the control group (p < 0.04).

A stand-alone, first in-vivo, validation experiment by ^19^F-MRS, involving oral administration of **c1a** to a rat was then carried with 1600 repetitions (800 s) per scan to test sensitivity following oral dosage of the tracer, with encouraging results (Supplementary figure S3). This was followed by replicate experiments in 6 animals (3 controls and 3 with prior dosing of an 11β-HSD1 inhibitor candidate). In all 6 animals the in vivo ^19^F-MRS spectra of the liver showed the two signals of **c1a** and **c1b** separated by ≈1 ppm, (Fig. 6a, representative). Of note the signals were much weaker in intensity than those of the isofluorane anaesthetic.

For the group dosed with the inhibitor candidate (Merck 544), the ^19^F-MRS signal of the substrate (**c1a**) was lower than for the control group (p < 0.04) (Fig. 6b). Total ^19^F-MRS signal (**c1a** + **c1b**) of the inhibitor group was also lower (p < 0.05), but inhibition was not detectable from **c1b** ^19^F-MRS signals or the ratio of substrate to product.

## Discussion

These data provide novel evidence that ^19^F-MRS discriminates keto and hydroxy steroidal compounds and may be used to develop tracers to quantify individual reactions in drug metabolism, such as those related to the activity of dehydrogenase/reductase enzymes such as 11β-HSD1, or indeed other steroid hydroxysteroid dehydrogenases. This provides valuable insight over and above measurements of drug and metabolite concentrations in tissues *in vivo*^24–28^.

Mono-fluorinated tracers were evaluated initially, using dexamethasone and 11-dehydrodexamethasone, which can be distinguished readily in standard ^19^F-NMR. Dexamethasone is licensed for human use and hence offered a rapid route to translation. As anticipated, signals in general were broader in MRS experimental settings than in NMR due to matrix effects and lack of sample spinning. Nonetheless in the biological matrices examined ex vivo, blood and perfused liver, the ^19^F-MRS signals for dexamethasone and 11-dehydrodexamethasone could be distinguished albeit being even broader than in solvent alone. Peak broadening can be attributed to a number of factors, including association of the tracer with larger molecules in the matrix or interaction with iron, affecting T2* relaxation^35^. This raised the limit of detection (approximately four-fold higher in blood than in organic solutions) due to a detrimental effect on signal/noise. These data also confirmed that ^19^F-MRS signal was linearly related to the total fluorine content within the scanned volume. Importantly the individual intensity of the ^19^F-MRS signal in biological matrix (blood and liver) has the same proportionality vs concentration for the keto and hydroxy tracer forms interconverted by catalytic action of 11β-HSD1, consistent with the ^19^F-MRS signal being proportional to the number of magnetically equivalent fluorine nuclei present in the tracer.

While detection of mono-fluorinated steroids was feasible using a 7T preclinical scanner, on a 3T clinical scanner, however, adequate sensitivity for detection could not be achieved in vivo in humans even after administration of a single high dose of dexamethasone. The signal to noise ratio increases with the B_0_ (magnetic field), with a scaling factor of 4.4 between 3 and 7T instruments offering future possibilities for improvement. Difficulties in detection could also potentially could be overcome by polyfluorinated tracers, should they be licensed for human use. However it is worth also noting that here, using licensed doses of dexamethasone, the circulating concentrations of dexamethasone achieved were substantially lower than the concentrations of other fluorine-containing drugs detected successfully by MRS. Indeed, although doses of dexamethasone used here were as high as might reasonably be used in healthy volunteers, they do not approach the doses of other (often poly-fluorinated) drugs used for previous ^19^F-MRS studies (> 500 mg^22,25,28^). The limited scope to further increase dosing of monofluorinated steroids was unlikely to yield the sensitivity gains required. The detection of (and resolution between) ^19^F-MRS signals from structurally very similar tracer and metabolite, achieved using the readily available and suitable for human fluorinated molecule dexamethasone and on a clinical scanner set up, was still a promising finding particularly in view of the low LOD_F_ measured using relatively short acquisition times.

Therefore, to address future potential, the use of poly-fluorinated non-steroidal tracers in conjunction with a more powerful 7T pre-clinical scanner was investigated. For comparative purposes, we introduced the use of LOD_F_ (limit of detection adjusted for total fluorine content, expressed in moles of fluorine) rather than the LOD (limit of detection as tracer concentration) usually reported in the literature^20^ to account for both the number of equivalent nuclei per molecule and the total amount of fluorine within the coil volume of detection. Using feasible scanning times (< 20 min), an LOD_F_ of 0.250–0.450 μmoles of fluorine for molecules in the library was consistently achieved in organic solutions. This is an order of magnitude improvement compared with the LOD_F_ of ~ 5 μmoles measured with the 3T scanner and highlighted the increased challenge of sensitivity on experiments at 3T field that is the more common field strength for clinical scanners.

The improved sensitivity gained with the polyfluorinated tracer, used on a 7T scanner, allowed the ^19^F-MRS signal to be readily detected in rat liver in vivo*,* using moderate weight matched doses and while achieving circulating levels much lower than the isoflurane used as anaesthetic. However, the fact that the fluorine nuclei were more distant in the polyfluorinated molecule from the keto/hydroxy group than in dexamethasone meant that the ^19^F-MRS signals were less shifted by the magnetic environment change and were less readily discriminated between enzyme substrate and product. Accordingly, spectral modelling analysis was used to measure ^19^F-MRS peaks from the substrate and product of 11β-HSD1. In blood, the modelling procedure for signals of similar magnitude was accurate at near LOD_F_, with an observed signal variation between scans of ≈7%. The procedure was similarly useful in monitoring activity in excised liver at mid-high tracer content. However, increased broadening of peaks and greater difference in signal intensities may render it less accurate for in vivo data acquisition when using very low tracer dosages or when looking for small differences in abundance. Thus precision would need assessed in workflows designed to measure pharmacodynamics for the 11β-HSD1 target for which enzyme inhibition of only 50% (as that of Merck 544) may be sufficient to elicit the desired efficacy^6^. Nonetheless we did detect both tracer and metabolite in all animals dosed with tracer **c1a,** proving that ^19^F-MRS can be used to measure signals from closely related tracer/metabolite pairs originated by oxidoreductase activity.

A single dose of the 11β-HSD1 inhibitor Merck 544, (an established inhibitor of the rat enzyme in vitro^12^) was administered which altered the ^19^F-MRS signals in vivo in rats, by unexpectedly reducing the overall abundance of **c1a** in the liver, whereas a difference in the amount of metabolite, **c1b**, formed was not observed. This paradox could be a result of a drug-drug interaction affecting the bioavailability or clearance of **c1a** and provides insight into the pharmacodynamics of this specific drug. Demonstration of the ability to detect tracer and metabolite in the specific tissue of interest by ^19^F-MRS highlights the value of having in vivo measurements when extrapolating candidate inhibitors from the *in* vitro setting and the potential of the approach. There are significant advantages of knowing tissue specific pharmacodynamics in vivo versus solely whole organism exposure studies by autoradiography.

This work provides a basis for future development of in vivo tracers, in particular defining the likely LOD_F_ and substrate/product peak separation required. However it was limited in the number of potential fluorinated enzyme substrates available. The number of human subjects tested was small but sufficient to establish that increases in sensitivity were required for translation to humans and the use of realistic doses. Preparation of polyfluorinated derivatives of keto-steroids (e.g. based on cortisone or 11-dehydrocorticosterone) may offer a potential route ahead, but these syntheses are challenging. To be useful, and for the signal to be additive, the synthesis must yield steroids containing multiple magnetically equivalent fluorine atoms close enough to the C-11 position so the NMR shift is sufficient for *in-vivo* MRS signal resolution. Non-steroidal compounds are more likely to provide a suitable skeleton for polyfluorinated tracers. Indeed some further members of the library investigated here have more than 3 magnetically equivalent fluorines, and some had greater separation of ^19^F-MRS signal between keto and hydroxy forms (unpublished data) but these were not substrates for 11β-HSD1. Thus a more extensive screen for such compounds, or modification of the lead compounds identified here, may provide suitable tools in future.

Technological advances will also benefit this area of study. Since a simple pulse-acquire pulse sequence was used here, the SNR in this work was mainly determined by the number of acquisitions. Future use of cryocoils has the potential to increase SNR by a factor of ~ 3 for rats, but the advantages are much lower for larger coils as used for human studies. Under-sampling methods would result in lower SNR but scanning with phased-array coils that cover a larger sample volume in the organs of larger animals may result in enhanced sensitivity compared with rodents and dual tuned coils also offer novel possibilities^36^. Also use of F–H decoupling where appropriate (in combination to general NMR sequence optimization) could be useful to enhance ^19^F-MRS *in-vivo* sensitivity^37^ in conjunction with higher field instruments.

As higher field strength MR is introduced into medical and large animal practice, even monofluorinated compounds may become suitable for tracer use^38^. Indeed commercial MRI systems of up to 21T are now available and 9.4T magnets are increasingly being used for preclinical scanning^39^. However unless sensitivity can be enhanced to the point of measuring mono-fluorinated steroids, a hurdle remains in the expense of toxicology studies required for use in humans that may limit the utility of ^19^F-MRS tracer studies to the pre-clinical arena.

^19^F MRS offers an approach to monitor metabolism of steroids by 11β-HSD1. Further single reaction oxidoreductase metabolic steps could thus be monitored in a tissue specific manner, but the approach is currently limited by sensitivity. Advances in MRS technology, such as increased magnetic field strength, optimised specific MRS acquisition sequences or the availability of polyfluorinated enzyme substrates may open doors for in vivo pharmacokinetic/pharmacodynamic studies, investigation of pathology and improved understanding during in vitro to in vivo translation.

## Methods

### Fluorinated tracers and chemicals

11-Dehydrodexamethasone was from Steraloids (Newport, Rhode Island, USA) and dexamethasone for pre-clinical studies from Alfa-Aesar (Heysham, UK). Dexamethasone tablets for clinical use were from Essential Generics (Egham, UK). 2-(Phenylsulfonyl)-1-(4-(trifluoromethyl)phenyl)ethanone (Fig. 1c, compound **c1a**) was originally provided by Wyeth (now Pfizer, Sandwich, UK); subsequent batches were synthesised in-house as previously described^34^ (Supplementary information). All other non-steroidal polyfluorinated keto compounds (Fig. 3) were from Apollo Scientific Ltd (Stockport, UK), with the exception of methyl 6-(trifluoromethyl)-nicotinoyl acetate (**c6a**) and 4,4’-bis(trifluoromethyl)benzophenone (**c8a**) which were from Marshalton Research Laboratories (King, North Carolina, USA). Corresponding hydroxyl metabolites were synthesised in house by reduction with sodium borohydride (Supplementary information). The selective 11β-HSD1 inhibitor Merck 544 (3-adamantan-1yl-6,7,8,9-tetrahydro-5H-[1,2,4]trazolo[4,3-a]azepine)^40^ was from Enamine Ltd (Kiev, Ukraine). All other chemicals were from Sigma-Aldrich (Poole, UK) and used without further purification unless otherwise stated. Solvents (HPLC grade where appropriate) were from Sigma-Aldrich, Fisher Scientific (Loughborough, UK) or Rathburn (Walkerburn, UK).

### Library of polyfluorinated substrates for 11β-HSD1

Putative polyfluorinated non-steroidal keto tracers (Fig. 3, compounds **c1a** to **c12a**) were selected by consideration of the published literature^41,42^ and structural similarity with the library lead, compound **c1a**^34^ and screened for inhibition of cortisone reductase activity. Tracers **c1a** to **c12a** (or vehicle) were added to HEK293 cells stably transfected with either human *HSD11B1* or rat *Hsd11b1* and inhibition measured in duplicate using a scintillation proximity assay as described^43^. Percentage inhibition was determined relative to vehicle control and IC_50_ determined using a four parameter logistic equation.

### Structural characterisation of steroidal and polyfluorinated tracers

NMR spectra (^1^H, ^13^C (decoupled) and ^19^F (decoupled)) were acquired using an ARX250 Bruker BioSpin NMR spectrometer (Billerca, USA) and referenced to a solvent peak (CDCl_3_ or methanol). (Supplementary Figure S1).

### Measurement of steroidal and polyfluorinated tracer ex vivo by ^19^F-MRS at 7T (pre-clinical scanner) in phantoms

A 7T pre-clinical scanner (Agilent Technologies, Yarnton, UK) fitted with a 400 mT/m gradient set and with a 30 mm diameter round surface coil for resonance frequency (RF) transmission and signal reception was used (approximate penetration depth of 15 mm). The scanner was tuned to the RF of ^19^F (280 MHz). Fluorinated keto tracers (including 11-dehydrodexamethasone and **c1a**) and their hydroxy metabolites were scanned in glass vials (sample volumes 1–20 mL) in organic solvent (chloroform) or whole blood (20:1 in 0.5 M ethylenediaminetetraacetic acid). Care was taken so all the sample volume was contained within the sphere of detection. To determine the linearity of the relationship between signal intensity and concentration and the limit of detection, samples were prepared by serial dilution in chloroform (5 mL) or whole blood, the latter spiked with tracer stock solutions (8–10 mg/mL prepared in chloroform, chloroform/dimethylsulphoxide (DMSO) 9:1 or DMSO). A range of concentrations of 11-dehydrodexamethasone in 5 mL was scanned using 50–3200 repetitions per spectrum (25, 100, 400 and 1600 s scan time). Scans were carried out at room temperature without stirring or spinning. When using blood as the matrix, samples were occasionally shaken between scans. Simple pulse-acquire scans were acquired with T_R_ of 0.5 s and with 50–3200 repetitions per spectrum, as indicated in Results.

### Measurement of dexamethasone in phantoms by ^19^F-MRS at 3T (clinical scanner)

A 3T Siemens Verio scanner (Siemens Healthineers, Erlangen, Germany) with a circularly polarized ^19^F-tuned transmit-receive flexible surface coil (180 mm × 244 mm, Rapid Biomedical, GmbH, Rimpar, Germany) was used for scanning phantom solutions of dexamethasone and 11-dehydrodexamethasone prepared in methanol (20 mL in glass vials). Saline bottles were used to load the coil and the phantom was placed between the loading bottles and the coil. Spectra ranging from 64 to 640 averages were used to measure LOD_F_ and signal-to-noise versus tracer content using pulse-acquire scans. T_R_ of 0.5, 1 and 1.5 s were tested and the shortest one that did not affect signal to noise used.

### Measurement of dexamethasone in human liver by ^19^F-MRS at 3T (clinical scanner)

With local ethical approval (South East Scotland Research Ethics Committee 01) and written informed consent, three healthy male volunteers (ages 24, 26 and 32 years) were recruited by advertisement. The study was performed in accordance with relevant guidelines/regulations, and in accordance with the Declaration of Helsinki. Inclusion criteria were age 16–60 years. Exclusion criteria were known hypersensitivity to dexamethasone or contraindications to MRI scanning; glucocorticoid therapy in the previous 3 months; diabetes mellitus; body mass index > 40 kg/m^2^; alcohol intake > 28 units/week; renal, thyroid or liver dysfunction on biochemical screening; history of dyspepsia or peptic ulcer disease; history of, or current treatment for, mental illness; pregnancy or lactation. Volunteers attended in the morning. A 20G IV cannula was inserted into the right antecubital fossa for serial venous sampling for *ex-vivo* measurement of dexamethasone and 11-dehydrodexamethasone (by liquid chromatography tandem mass spectrometry (LC–MS/MS)). Dexamethasone was administered orally (volunteer 1, 10 mg; volunteer 2, 12 mg; volunteer 3, 14 mg) with the aim of detecting a signal for dexamethasone in liver and determining the lowest dexamethasone dose which could be detected.

Volunteers were then scanned in the Verio 3T scanner as for in vitro phantoms. The flexible surface coil was placed directly over the liver, wrapping around the body areas of the liver such that the optimal excitation happened in the middle of the liver. A 90 degree excitation 100 µs block pulse was used with a vector size of 128, and spectra obtained at isocentre with a second order semi-automated shim applied before acquisition, with a non-localised FID, echo time (T_E_) of 0.23 ms, and a bandwidth of 2000 Hz. T_E_ denotes time from the centre of the RF pulse to the start of acquisition. The system was set to nominally generate a 90 degree flip angle, which it should do at the “optimal” excitation point as dictated by the circumference of the coil. Pulse calibration was not possible and higher flip angles were tried empirically without any obvious increase in signal. No phase cycling was used, with an acquisition bandwidth of 10,000 Hz. T_R_ of 1500 ms and T_E_ of 0.23 ms were used with 400 repetitions (scan time 10 min) per spectrum. Anticipating a transient rise in hepatic dexamethasone concentrations within two hours post administration, six 10 min scans were conducted at intervals from each participant between 30 and 100 min post dexamethasone administration. In two of the three subjects, a vial with dexamethasone solution in methanol was placed between the coil and patient liver to confirm coil operation and ^19^F-MRS signal detection prior to oral dexamethasone administration. The phantom was then removed, dexamethasone administered, and in vivo scanning of the liver commenced.

### LC–MS/MS analysis of dexamethasone

Dexamethasone and 11-dehydrodexamethasone were measured by liquid chromatography tandem mass spectrometry (LC–MS/MS). Stock solutions of analytes and internal standard were prepared (10 µg/mL in methanol) and stored at − 20 °C. Standards were prepared on the day of analysis by serial dilution of stock solutions. A standard curve was prepared representing a concentration range of 0–300 ng/mL for all analytes. 4,6α,21,21-Dexamethasone (d4-dexamethasone, internal standard, 15 ng; C/D/N/ Isotopes. Quebec, Canada) was added to plasma (200 µL) and standard curve samples. Calibration curves and samples were processed by solid-phase extraction (Sep-Pak® C18, 200 mg cartridges (Waters, Wilmslow, UK), conditioning with 5 mL methanol, equilibration 5 mL water followed by sample loading, washing with 5 mL water and analyte elution with 2 mL methanol). Eluates were dried under nitrogen (60 °C), resuspended in water (200 µL) and then extracted with ethyl acetate (2 mL). The supernatant was dried and dissolved in mobile phase. Injection volume was 10 µL. Analysis was performed on a Waters Acquity™ UPLC with autosampler (10 °C), coupled to an AB Sciex QTRAP® 5500 mass spectrometer (Warrington, UK), and operated with Analyst Software version 1.5.1. Separation was achieved on a SunFire™ C18 column (150 × 4.6 mm, 5 µm; Waters) at 20 °C, with a linear gradient from 60:40 to 45:55 (acetonitrile with 0.1% formic acid (FA): water with 0.1%FA) at a flow rate of 1.5 mL/min with a total run-time of 6 min. Ionisation was performed in positive electrospray mode with curtain gas 20 psi, collision gas medium, source temperature 500 °C and source gas 40 psi. Mass transitions of protonated ions monitored were (Declustering Potential, DP; Collision Energy, CE; Cell Exit Potential, CXP): dexamethasone *m/z* 393 → 373 (DP 71; CE 11; CXP 16); 11-dehydrodexamethasone *m/z* 391 → 253 (DP 71; CE 27; CXP 12); d4-dexamethasone m/z 397 → 377 (DP 51; CE 11; CXP 16). Intra-assay precision and accuracy respectively of the assay were 10.1% and 13.1% for dexamethasone and 7.7% and 0.3% for 11-dehydrodexamathasone in the relevant concentration range (n = 6 replicates), with a lower limit of quantitation of 0.25 ng/mL, measured against a 1/x weighted calibration line.

### Measurement of polyfluorinated tracer in perfused rat liver by ^19^F-MRS at 7T (pre-clinical scanner)

In an initial ex vivo experiment, 32.8 mg **c1a** was dissolved in 4 mL DMSO:PEG_400_ 1:1 and diluted in Krebs buffer (1 L) to a final concentration of 100 µM before perfusion into an excised rat liver (further details below) via the portal vein at 25–30 mL/min for 30 min. Subsequent scanning was carried out with 800 repetition (400 s) per spectrum (11 scans acquired) with the vial containing excised liver placed inside the coil.

### Measurement of polyfluorinated tracers in rat liver in vivo by ^19^F-MRS at 7T (pre-clinical scanner)

Male Wistar rats (Harlan Olac, Bicester, UK), weighing between 415 and 515 g were studied under license from the UK Home Office and approved by the Animal Welfare and Ethical Review Board committee, University of Edinburgh. All procedures were performed under the UK Animals (Scientific Procedures) Act, 1986 and carried out in compliance with the ARRIVE guidelines. Rats were anesthetized with 4% isoflurane (Merial Animal Health Ltd., Harlow, UK), followed by 1.2–1.4% in oxygen enriched air for maintenance, and placed in an MRI compatible rat holder (Rapid Biomedical GmbH, Rimpar, Germany). To minimize the scanning time and maximise the signal-to-noise ratio of the ^19^F spectra, no localization was applied. Positioning of the animal and coil were initially checked by the acquisition of fast gradient echo scout images (T_R_ 30 ms, T_E_ 2 ms, FOV 50 × 50 mm, matrix 128 × 128, and slice thickness 3 mm) in 3 orthogonal orientations (Supplementary Figure S4). The homogeneity of the magnetic field was optimized using the ^1^H signal before the coil was tuned to the resonance frequency of ^19^F (280 MHz). Untriggered ^19^F-MRS spectra using pulse-acquire sequence were acquired with following parameters: T_R_ 0.5 s; 800 signal averages (400 s) per spectra unless otherwise indicated; spectral width 20,000 Hz centred near the tracer signal. Regarding the flip angle, a 100 µs block pulse was used. This gave the best SNR when using a 0.5 s TR. Because a surface coil was used, the flip angle varies with the distance from the centre of the coil, so an accurate value cannot be given. After scanning was completed, rats were killed by cervical dislocation while still under anesthesia.

Preliminary validation used a range of concentrations (10–20 mg/kg of **c1a** and 20 mg/kg of **c1b** with up to 800 s (1600 repetitions) per spectrum. Furthermore mixtures of **c1a** and **c1b** in proportions 1:0, 4:1, 1:1, 1:4 and 0.1 were scanned and the signal intensities measured. The resultant deconvoluted data were compared against the predicted value. ^19^F-MRS experiments to assess pharmacodynamics of tracer **c1a** and to test the effect of 11β-HSD1 inhibition used **c1a** at 15 mg/kg (administered by gavage, dissolved in olive oil:PEG_400_:DMSO 95:5:5 as vehicle at 5 mg/mL). After overnight fast, rats were gavaged with inhibitor (Merck 544, 5 mg/mL in vehicle) at 30 mg/kg (n = 3) or vehicle (n = 3, weight matched as with inhibitor) then anesthetized and positioned prone on top of the surface coil and scout images obtained as above. The animals were removed from the scanner and dosed with tracer **c1a**. The tracer was administered 35–40 min after Merck 544. The animals were repositioned into the holder (taking care to avoid including stomach or gut within the coil inner detection volume), scout images re-taken and the instrument switched to ^19^F-MRS mode. Spectra (400 s per spectrum) were acquired sequentially from 25 (± 2) minutes until 83 (± 2) minutes post **c1a** gavage.

### Data analysis

#### 7T-MRS signal processing

^19^F-MRS spectra were processed using jMRUI^44^ AMARES algorithm. For phantom work, line broadening of 20 or 50 Hz was applied as it gave more reliable signal intensity results. For animal studies, 50 Hz line broadening was applied unless stated otherwise. The offset spike was removed; when present, the highest isofluorane peak was used as a reference for signal position and isofluorane peaks were filtered out using the HLSVD algorithm before peak fitting and signal integration. If no isofluorane was present, a 0 ppm value was given to the centre of the spectrum. Overlapping tracer/metabolite ^19^F-MRS peaks were resolved by using soft constraints on signal position and line width. Prior knowledge parameters were based on the values obtained from ^19^F-MRS experiments when only one of the signals was present.

#### Statistical analysis

^19^F-MRS data are given as mean ± SEM and compared by repeated-measures ANOVA, with Fisher *post-hoc* tests as appropriate, with significance accepted at p < 0.05.

## Supplementary Information

Supplementary Information.
