# Supplementary material for: Non-invasive in vivo assessment of 11β-hydroxysteroid dehydrogenase type 1 activity by 19F-Magnetic Resonance Spectroscopy
Source: Sci Rep. 2022 Sep 29;12:16268. doi: 10.1038/s41598-022-18740-5 (PMC9523021; doi:10.1038/s41598-022-18740-5)
Supplement: Supplementary file 1 — Supplementary Information. [file 41598_2022_18740_MOESM1_ESM.pdf]

Supplementary Figure S1

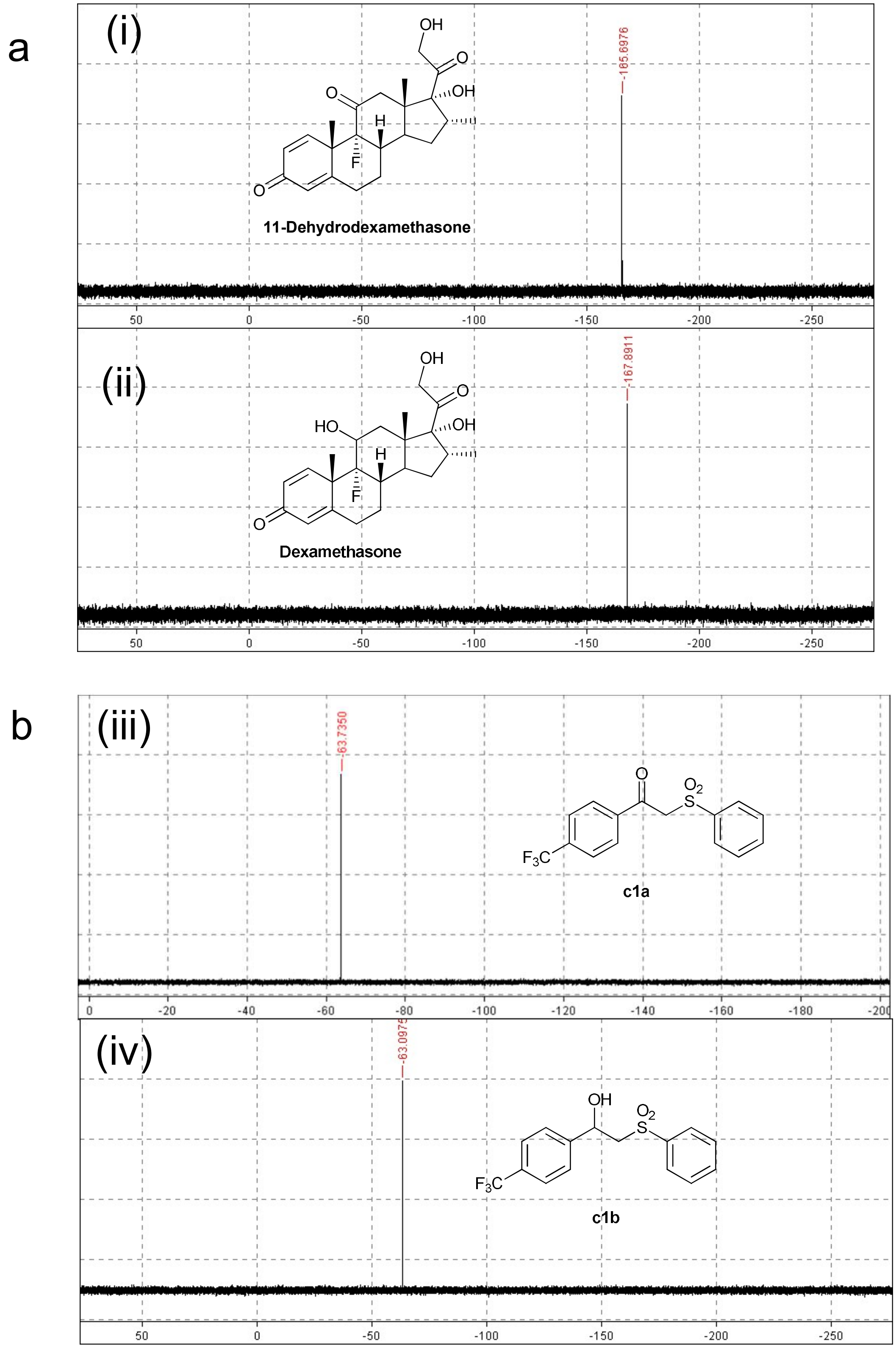

Supplementary Figure S1: Distinct resonance frequencies of keto substrates an hydroxy products of 11β-hydroxysteroid dehydrogenase 1

^{19}F-NMR in $CDCl_3$ (ppm values relative to solvent peak) measured on a Bruker NMR (^{1}H 250MHz) spectrometer. (a) (i) monofluorinated keto steroid 11-dehydrodexamethasone and (ii) its hydroxy metabolite dexamethasone, showing a 2.2 ppm difference. (b)) trifluorinated tracer (iii) **c1a** and its metabolite (iv) **c1b,** showing a 0.6 ppm difference.

Supplementary Figure S2

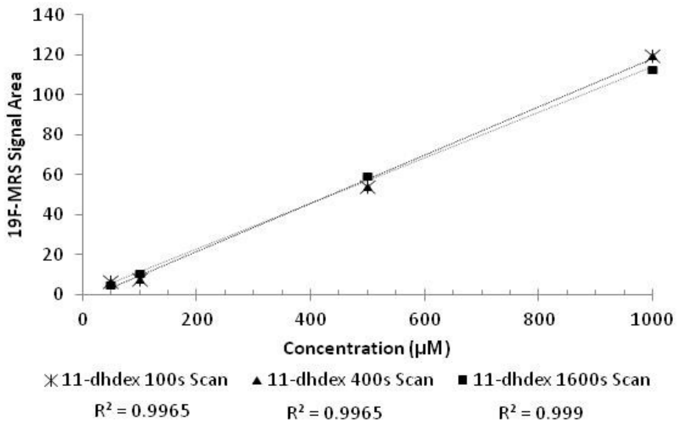

Supplementary Figure S2: Linearity of ^{19}F-MRS signal area vs concentration

^{19}F-MRS signal vs. concentration (50µM to 1000µM) for 11-dehydrodexamethasone (11-dhdex) at 100s, 400s and 1600s acquisition time,.

Supplementary Figure S3

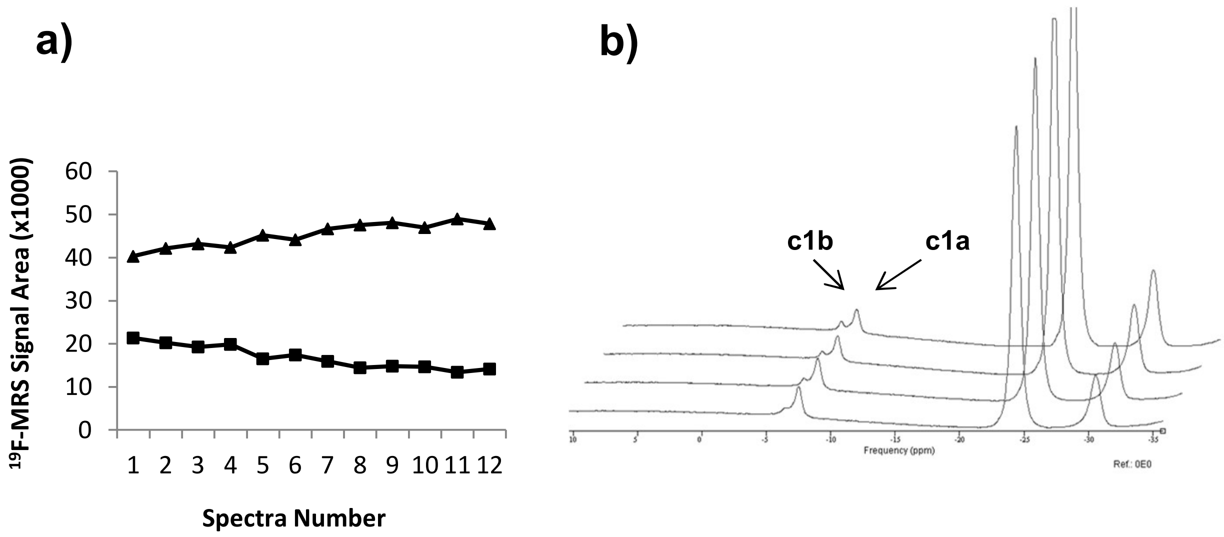

Supplementary Figure S3: Ex-vivo (perfusion) and in-vivo conversion of keto compound c1a to hydroxy metabolite c1b detected by ^{19}F-MRS scan liver in rat.

(a) Ex-vivo scan of liver. Signal areas vs spectrum on the serial scans of the pilot experiment, each for 400 s, beginning ~20 minutes after the end of a 30 minutes perfusion with **c1a** 100 µM solution. **c1b** was formed rapidly during the perfusion with **c1a** and continued to accumulate during scanning.

(b) Sequential (front to back) *in-vivo* in rat liver 19F-MRS spectra after gavage of 8mg **c1a** and with double time acquisition (1600 repetitions, 800 seconds per spectrum). Signals of **c1a** and **c1b** formed *in vivo* appear clearly, upfield from the more intense isofluorane peaks, and change over time consistent with reduction of **c1a** to **c1b**. 0 ppm was assigned to centre of spectra range.1600 repetitions per spectra were used to enhance signal-to-noise and assist on visual positioning of signal.

Supplementary Figure S4

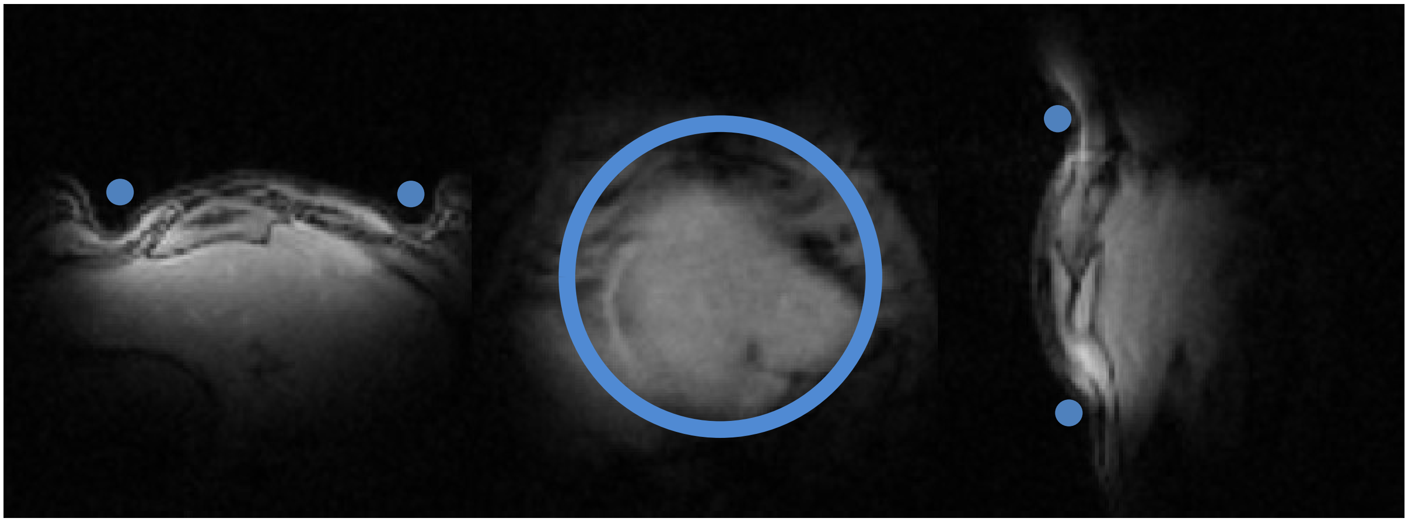

Supplementary Figure S4
Images showing the positioning of a rat undergoing magnetic resonance spectroscopy imaging. 3 orthogonal slices (axial, coronal, sagittal) were acquired at the centre of the surface coil, showing the liver and indicating the coverage of the coil. The position $^{19}F/^{1}H$ radiofrequency coil is indicated by the blue circle and cross sections.

Supplementary Information

Chemical synthesis

Synthesis of non-steroidal polyfluorinated keto tracer c1a

2-(Phenylsulfonyl)-1-(4-(trifluoromethyl)phenyl)ethanone (compound **c1a**, Figure 1c) was originally provided by Wyeth (now Pfizer); subsequent batches were synthesized in-house as previously described [1, 2] with modified purification. In brief, 2-bromo-1-(4-(trifluoromethyl)phenyl)ethanone (267.04 mg, 1 mmol) was dissolved in DMF (4 mL). Sodium benzenesulfinate (164.16 mg, 1 mmol, 1eq.) was added in one portion, and the mixture heated (110 ^{o}C, 2.5 hours) with stirring. Vacuum evaporation gave a dark orange, oily residue. The oil was partitioned between ethyl acetate and water, the organic layers combined, washed with saturated aqueous NaCl, dried with $MgSO_4$, filtered and evaporated to give a crude orange solid (91% yield). Two crystallisations from hot ethyl acetate/hexane (1:4) generated bright white needles (30% final yield). These were confirmed as the desired product by ^{1}H and ^{13}C-NMR and by LC-MS/MS analysis yielding the protonated molecular ion with mass of *m*/z 329 [M+1]. ^{19}F-NMR in $CDCl_3$ showed a single signal at 63.73 ppm (Supporting figure S1b(iii))

Synthesis of non-steroidal polyfluorinated hydroxy metabolites

To prepare the hydroxy metabolite of compound **c1a** (i.e. 2-(phenylsulfonyl)-1-(4-(trifluoromethyl)phenyl)ethanol, compound **c1b**, Figure 1c), **c1a** (99 mg, 0.3 mmol) was dissolved in ethanol/dichloromethane 1:1 (3 mL). Sodium borohydride (15.13 mg, 0.4 mmol, 5.3 eq.) was added in one portion and stirred (room temperature, 2h). The reaction was quenched with hydrochloric acid (700µl, 0.5M), diluted with water (6 mL) and extracted with dichloromethane (12 mL x3). Organic extracts were combined, washed with brine, dried with MgSO4 and filtered. TLC (ethyl acetate/hexane 1:4) showed only one spot (Rf=0.38). Solvent was removed under vacuum yielding a pale yellow oil. A solid was precipitated by addition of ethyl ether and dried under vacuum to give 87mg (88% yield). The compound was used without further purification. 1H and 13C-NMR of the solid agreed with the expected product. 19F NMR in CDCl3 gave a single signal at 63.09 ppm. (Supporting figure S1(iv)).

[1] J. Xiang, M. Ipek, V. Suri, W. Massefski, N. Pan, Y. Ge, M. Tam, Y. Xing, J.F. Tobin, X. Xu, S. Tam, Synthesis and biological evaluation of sulfonamidooxazoles and beta-keto sulfones: selective inhibitors of 11beta-hydroxysteroid dehydrogenase type I, Bioorganic & Medicinal Chemistry, 15 (2005) 2865-2869.

[2] J. Xiang, M. Ipek, V. Suri, M. Tam, Y. Xing, N. Huang, Y. Zhang, J. Tobin, T.S. Mansour, J. McKew, beta-Keto sulfones as inhibitors of 11beta-hydroxysteroid dehydrogenase type I and the mechanism of action., Bioorganic & Medicinal Chemistry, 15 (2007) 4396-4405.
